# Goat lactation research as a gateway for the development of the dairy goat industry

**DOI:** 10.1093/af/vfad005

**Published:** 2023-06-14

**Authors:** Noemí Castro, Aridany Suarez-Trujillo, Marta Gonzalez-Cabrera, Lorenzo E Hernandez-Castellano, Anastasio Argüello

**Affiliations:** Animal Production and Biotechnology Group, Institute of Animal Health and Food Safety, Universidad de Las Palmas de Gran Canaria, 35413 Arucas, Spain; Department of Animal Science, Berry Colle, Mount Berry, GA 30149; Animal Production and Biotechnology Group, Institute of Animal Health and Food Safety, Universidad de Las Palmas de Gran Canaria, 35413 Arucas, Spain; Animal Production and Biotechnology Group, Institute of Animal Health and Food Safety, Universidad de Las Palmas de Gran Canaria, 35413 Arucas, Spain; Animal Production and Biotechnology Group, Institute of Animal Health and Food Safety, Universidad de Las Palmas de Gran Canaria, 35413 Arucas, Spain

**Keywords:** apocrine, genetic diversity, goat, mammary gland

**Implications**

- Milk somatic cell count is not a suitable indicator for goat’s udder health.
- Milking management protocols should be based on udder morphology characteristics which are influenced by genotype.
- Research gaps related to how perform a quick subclinical mastitis test on dairy goat farms.

## Introduction

The importance of dairy goats has increased in the last decades because of the higher demand for dairy goat products for human consumption. The dairy goat industry is constantly expanding, and the global goat herd has risen exponentially during the last decade compared to sheep and cattle. Goat milk represents 2.3% of global milk production, higher than sheep (1.3%). Some key factors for the success of the goat industry are the greater breed diversity (>500 breeds) and their capacity to adapt to harsh conditions in most environments. Goat milk is gaining interest due to its organoleptic properties and lower allergenic components in developed countries. These key factors make dairy goat production a unique alternative in developing countries. Dairy goats with high-yielding genotypes are mainly located in Europe. In addition, dairy goats fit the UN 2030 Agenda for Sustainable Development, mainly because they reinforce the role of women in agriculture and their economic independence. Goats can be easily owned and maintained, and their milk is an essential source of nutrition for children. For these reasons, the study of goat lactation is an important issue nowadays.

## Lactation, Milking, and Mammary Gland

The diversity of goat breeds impacts multiple variables, such as lactation period length, milk yield, and lactation persistency. Thus, the lactation length in goats can differ not only depending on the breed but also on the type of management, lasting from 7 to 10 months and peaking between 4 and 8 weeks in most breeds (Salama et al., 2005). Moreover, lactation persistency greatly variates depending on genetic traits and the shape of the lactation curve (Arnal et al., 2018). Milk yield and composition in dairy goats also vary depending on the breed; thus, the average milk ranges from 700 Kg (Arnal et al., 2018), 3.3% fat and 2.9% protein in Saanen or Alpine to 550 Kg, 3.94% fat and 3.9% protein in Majorera breed (adjusted to 210 days records) (Fresno et al., 2009). The variation in lactation length and persistency between breeds and management systems makes comparing scientific studies and industry standardization extremely difficult.

The effect of milking management is highly important due to breed diversity. Although goats are machine milked in developed countries, hand-milking management is still used in developing areas. The selection of the milking parameters (vacuum pressure, pulsation rate, and pulsation ratio) is crucial to preserve the udder health and milk quality. Milking vacuum pressure commonly ranges from 38 to 42 Kpa, the pulsation rate should be from 90 to 120 pulse/min, and the pulsation ratio should be 60/40, however the breed is always a factor to be considered when selecting suitable milking parameters. Thus, in the case of Saanen goats 35–38 Kpa and 90–120 pulses/min or 42 Kpa and 90 pulses/min for Majorera goats have been reported as adequate milking parameters (Le Du and Benmederbel, 1984; Billon et al., 2005; Torres et al., 2013). Milking frequency highly depends on the technology used on the farm. It is common twice daily milking in highly technological farms, obtaining 15% more milk (Komara et al., 2009). However, small-scale farms usually milk once daily because the increase in milk yield is insufficient to cover the extra labor costs. In addition, in some dairy breeds adapted to milking once a day, the more frequent milking does not considerably increase milk yield (Capote et al., 2000).

The udder glandular parenchyma is responsible for milk synthesis and comprises tubule-alveolar glands. Connective tissue surrounds the glandular and provides support to the udder. The mammary gland anatomy and histology change throughout the lactation cycle and affect milk quantity and quality traits (Léiras et al., 2014). In the seventies, multiple research groups clarified the milk synthesis process by the caprine lactocyte. Nowadays, it is clear that the goat milk secretion process is mainly apocrine, quite different compared with cattle. What does this mean? It means that part of the lactocyte, with its content, is pinched off the cell and poured into the alveolar lumen, and those portions of the lactocyte are known as cytoplasmic fragments, which are highly present in the milk. This different milk secretion mechanism, merocrine (cow) versus apocrine (goat), could be the reason for the different consistency for somatic cells count (SCC) as a predictor of intramammary infections, which must be face in the next years by the scientific community.

In milk, somatic cells (**SCs**) are mainly neutrophils, lymphocytes, and macrophages. They are the surveillant immune system in the mammary gland, which means that SCs react by increasing their number and activity to protect the mammary tissue from infections. The use of SC count in milk as a quality standard in cows is extended worldwide, but the topic has a shaky history in goats. The California Mastitis Test is the on-farm standardized detection method for mastitis in dairy ruminants. The procedure detects clinical and subclinical mastitis and is based on the reaction between the DNA from SC with the testing reagent. The increase of SC as a response to mastitis increases the amount of DNA in milk, and the test will be more reactive. The problem in goats appears when an increased SC count in the milk is not concurrent with an infection. There are reports of increased SC in goats, to levels that are considered pathological in cattle, during the beginning and the end of lactation, during the estrous cycle, under stress, or as individual idiosyncrasy. In fact, Zeng and Escobar (1995) reported that milk with high SCC (over 1 × 10^6^ SC/mL) can be obtained from healthy goat udders. The larger number of SC in the milk of healthy goats makes the diagnosis of subclinical mastitis and the management of herd health difficult. The use of nobel techniques, such as proteomics looking for specific biomarkers in goat milk, should be reinforced, thus the identification and prevention may be improved. Thresholds have been set for SC count in goat milk in different countries and unset years after because there is no conclusive scientific evidence of its use in goat milk. That is the case in the United States, where the SC standard for goat milk increased from 1 × 10^6^ somatic cells/mL to 1.5 × 10^6^ somatic cells/mL (grade “A”), most likely because of the influence of not infectious factors on the SCC. In fact, despite some countries in Europe established a threshold in the past, currently the EU lacks an SCC level in the goat milk standards, most likely because of the cited uncertainties (Haenlein, 2002). However, in some places the dairy industry includes this variable as a requirement for goat milk reception.

**Figure 1.**
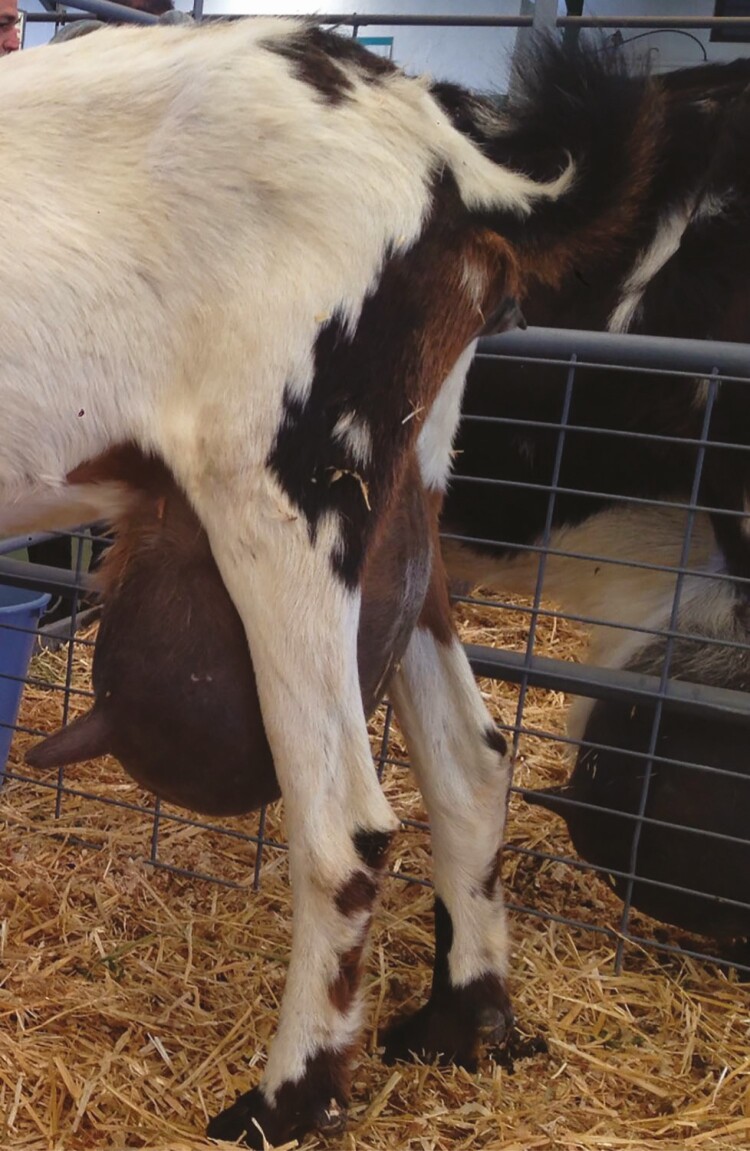
Teat insertion in Majorera dairy goats.

The breed, age, and lactation stage influence goats’ cisternal and alveolar partitioning of the udder. Cisternal milk in dairy cows represents 30% of the stored milk (Ayadi et al., 2004) and in dairy goats can reach at least 66% (Suárez-Trujillo et al., 2013). Goats have larger cisterns than cows, and the teat insertion is more horizontally and laterally in several breeds (Figure 1). These udder characteristics make the manual intervention during milking necessary to remove the milk below the teat insertion (Torres et al., 2013). Thus, in some goat breeds, machine-stripping milk (**MSM**) is considered the milk that requires manual intervention to be collected. As MSM fraction increases, the manual time intervention increases, which implies more labor costs. Furthermore, automatic cluster removers are not helpful in those breeds where manual intervention is required because once the cluster is removed, the milker must put it again, which is not recommended for preserving the udder’s health.

The goat mammary gland anatomy displays a large cistern compared to other ruminants such as ewes and cows. The large cistern allows goats to accumulate more milk between milkings than the other two species, and to be less dependent on oxytocin for milk let-down. Goats with a high milk yield and flow rate show no increase in oxytocin levels. According to this knowledge, the standard milking routine in dairy goat farms includes teat cleaning, foremilk stripping, immediate cluster attachment, machine milk removal, stripping machine milk removal, cluster removal, and teat dipping. However, recently it has been described that dairy goats present different milk let-downs based on other stimuli (González-Quirino et al., 2021).

## Conclusion

The exchange of knowledge about lactation from dairy cows to dairy goats seems to be not adequate because of the significant differences between these two ruminants. Additionally, the high variability in most traits, principally due to genetic diversity, makes further studies about mammary gland physiology, milk yield, and composition, especially those related to the SC counts, essential for a growing industry. Although a challenge for the coming years, developing a suitable subclinical mastitis test specific for dairy goats would be very helpful for goat farmers.

## About the Authors

**Noemí Castro** is Associate Professor for the Animal Science Department at Las Palmas de Gran Canaria University (ULPGC), Canary Islands, Spain. Presently, she coordinates de Animal Production and Biotechnology research team and the Master of Animal Health and Food Safety at ULPGC. She is the author or coauthor in over 96 peer-reviewed articles (JCR), over 30 articles in nonrefereed publications, 5 book chapters and over 150 abstracts in meetings. H-index 26 (clarivate). She is the associate editor of the Journal of Applied and Animal Research, and a frequent reviewer in different journals (JCR). She has been involved on the board of different International Scientific meetings, such as the XI International Conference on Goats, Small Ruminants Committee of the ADSA, being chair of the 2021 ADSA Annual Meeting. Her favorite research topics are colostrum quality, goat newborn management, immunity, milking, and milkability. She currently is President of the International Goat Association.

**Aridany (Ary) Suarez** is an Assistant Professor of Animal Sciences at Berry College. He obtained his bachelor’s degree in Veterinary Sciences, master’s, and PhD in Animal Health and Food Safety at the University of Las Palmas de Gran Canaria in The Canary Islands, Spain. After finishing his graduate programs, he worked as a Postdoctoral Research Associate at the Department of Animal Science at Purdue University. During this period, he participated in multiple studies focused on understanding the role of the circadian timing system on mammary gland physiology. Those studies were developed in dairy cows, mice, humans, and cell cultures. His current research focuses on the influence of periparturient parasite rise in sheep on colostrum and milk quality.

**Marta González Cabrera**, PhD Student in Animal Health and Food Safety program at the University of Las Palmas de Gran Canaria. I studied Veterinary Medicine at the same university, earning my bachelor’s degree in 2021. In 2022, I got a Master degree in Animal Health and Food Safety Institute (IUSA), defending my dissertation on the effect of intramammary application of bacterial lipopolysaccharides on dairy goats. Currently, I am working as a Predoctoral Researcher at Animal Production and Biotechnology group at the University of Las Palmas de Gran Canaria on account of a local government grant. As part of this group, I am investigating on immune development in newborn ruminants (calves, lambs, and kids); blood, milk, colostrum, and mammary gland proteomics; seasonal weight loss (feed restriction) on mammary gland and lactation physiology and milk quality; and biomarkers for mastitis detection.

**Lorenzo E. Hernandez-Castellano**. I studied at the *Universidad de Las Palmas de Gran Canaria* (ULPGC, Spain), where I obtained my Veterinary degree and my Master in Animal Health and Food Safety. I defended my PhD thesis at the ULPGC (2013) under a European Doctorate basis (*Doctor europeus*). During my PhD, I performed research stays in Portugal (4 months), Denmark (3 months), and United States (3 months). From 2014 to 2017 (42 months), I worked as a postdoctoral researcher at the University of Bern (Switzerland). After a few months as a postdoctoral researcher at the Department of Molecular Biology and Genetics (Aarhus University, Denmark), I was selected for an associate professor position at the Department of Animal Science (AU) where I worked until January 2021, as I got one *Ramón y Cajal* contract offered by the *Ministerio de Ciencia, Innovación e Universidades* (Spain). From February 2021, I am working at the ULPGC as *Ramón y Cajal* researcher.

**Anastasio Argüello** earned the degrees of Bachelor of Veterinary Science (1995) and a PhD (2000, Animal Science). After a postdoctoral period of research in the USDA (Beltsville, MD), he moved to the Animal Science Department of the ULPGC as Assistant Professor and finally got the full Professor with tenure in 2015. He is the editor-in-chief of the Journal of Applied Animal research and an expert in small ruminant production. She is the author or coauthor of 142 Publications from the Web of Science Core Collection and 40 articles in nonrefereed publications, 3 book chapters, manuals, and over 200 abstracts in meetings. H-index 28 according to Clarivate. Recently he organized the first International Scientific Meeting on Colostrum. His main research interests are colostrum, immunology, meat, milk, and cheeses.
